# The Persian Language Version of the Obsession With the COVID-19 Scale for Adolescents

**DOI:** 10.3389/fpsyt.2021.800982

**Published:** 2021-12-24

**Authors:** Faramarz Asanjarani, Monika Szczygieł, Amna Arif

**Affiliations:** ^1^Department of Counseling, Faculty of Education and Psychology, University of Isfahan, Isfahan, Iran; ^2^Department of Neurocognitive Psychology, Faculty of Pedagogy and Psychology, Institute of Psychology, Pedagogical University, Kraków, Poland; ^3^Department of Education, Social Sciences and Humanities, University of Management and Technology, Lahore, Pakistan

**Keywords:** COVID-19, obsession, mental health, adolescent, validation

## Abstract

**Objective:** The worldwide pandemic of coronavirus disease (COVID-19) has caused significant public health burdens and psychological dysfunctions. In this challenging time, adolescents require special care. The Persian version of the Obsession with COVID-19 Scale (OCS) for adolescents was developed to screen for dysfunctional obsession associated with the coronavirus during the global pandemic. The structure and internal consistency of the OCS were established.

**Design and Measures:** Although there are different language versions of the OCS, this is the first study to validate the psychometric properties of the OCS in Iranian adolescents. Seven hundred and nine students (369 girls) participated in the study. Demographic questions and the OCS were administered.

**Results:** The findings provided support for the existence of a unidimensional structure that met the criteria for configural, metric, and full scalar invariance across gender (girls and boys), inhabitancy (urban and rural), and infection experience (infected and non-infected). The OCS is short and highly reliable measurement. However, further research is necessary to establish the validity of the scale in Iranian population.

**Conclusions:** The development of such valid scales is an essential part of both research and practice during times of crisis, like a global pandemic. Diagnosis of pandemic related to obsessive thoughts in adolescents is needed as the COVID-19 pandemic is still ongoing and as experts point out, it can be expected that the effects of the pandemic will be observed in the coming years. The Persian version makes it possible to conduct international comparative research on the anxiety related to the COVID-19 pandemic.

## Introduction

### Impacts of COVID-19 on Daily Life and Mental Health

Since December 2019, the coronavirus epidemic, also known as COVID-19, originated from Wuhan, China, and spread rapidly worldwide (1). Scientifically, the virus is referred to as Severe Acute Respiratory Syndrome Coronavirus (SARS-CoV-2) (2). The most common COVID-19 symptoms are fever, fatigue, dry cough, myalgia, dyspnea, etc., and can develop 2–14 days after infection through respiratory droplets and close contact (1, 3). In this article, we will refer to this disease as COVID-19.

The rapid emergence of the COVID-19 outbreak worldwide has dramatically changed daily behavior, significantly impacting the population's physical, social, mental, and financial well-being (4). Across the world, individuals must navigate school closures, employment insecurity, and social behavior changes, which are likely to negatively affect their mental health and coping abilities (5, 6). Recognizing that increased stress can lead to maladaptive behaviors to cope with stress and anxiety, health professionals highlighted the need for all individuals to manage stress and maintain their mental health during this highly uncertain period (6, 7).

Some of the most common social problems of the COVID-19 outbreak are fear of contracting this highly contagious virus, fear of losing loved ones, the spread of misinformation about COVID-19, the lack of medical treatment, the lack of adequately equipped units to treat patients, problems with lock-out [e.g., prolonged home isolation and social distancing; (8) insecurity, fear of unemployment, loss of income, etc.], depression, anxiety, phobia, insomnia, trauma, etc., are also common consequences associated with the COVID-19 outbreak (9). As a result, an increase in the suicide rate is common during and after the pandemic, which has also been reported during the COVID-19 pandemic (10, 11). Studies indicated that mental health problems and mediators increase the risk of suicide (12). It has also been reported that the physical performance of individuals and immunological stabilities are related to psychological states (3). However, studies reported increased psychological problems and low quality of life across nations and professions (e.g., healthcare professionals to general people) (13).

While studies are increasingly focusing on the mental health effects of pandemics on adults, relatively few studies focus on the effects of pandemics on children and adolescents (14). A study conducted by Chen et al. (15) showed that COVID-19 affects symptoms of mental disorders, including obsessive-compulsive disorder (OCD), fear, hypochondria, depression, and neurasthenia in college students. Adolescents may struggle with thoughts of a pandemic by observing the changing life situation at home as well as at school. In this study, which is part of a larger cross-cultural study, we investigated the factor structure and reliability of the Obsession with COVID-19 Scale [OCS; (16)] for Iranian adolescents. Below we present our rationale regarding why it is essential to focus on the emotional sphere of adolescents.

### Pandemic and Adolescents

In times of pandemic, as in disasters, there is an increased risk of Post Traumatic Stress Disorder (PTSD), depression, and anxiety (17). In a survey of 8,079 Chinese adolescents aged 12–18 years, Zhou et al. (18) reported a high prevalence of depression (43%), anxiety (37%), and combined symptoms of depression and anxiety (31%). In addition, Odriozola-González et al. (19) reported that during the current pandemic, adolescents experience a new period of insecurity: worry about their relatives' health and work, the ubiquitous problem of death, sudden separation from friends, and school interruption. In a sample of universities in Spain, many students experienced moderate to extremely severe anxiety (21%) and depression (34%) during the first weeks of quarantine. Another psychological impact of the epidemic on adolescents is post-traumatic stress disorders that affect brain development. PTSD in children is associated with changes in fronto-limbic circuits that may contribute to increased threat reactivity and weaker emotion regulation (20).

Anxiety is also a common psychological problem experienced by adolescents during epidemics. According to Cao et al. (21), having a relative or acquaintance infected with COVID-19 was a risk factor for anxiety in a Chinese undergraduate student population (21). Studies also show that absenteeism in children and adolescents is associated with reduced physical activity, more screen time, irregular sleep patterns, and less appropriate diets (22).

Outbreaks may also be linked to increased suicide rates (23). Family confinement can trigger domestic violence during the COVID-19 pandemic. Some countries, such as France and Brazil, have reported an increase in reported domestic violence cases; children are at greater risk of abuse or neglect when they live in a home where there is domestic violence. It is reported that women and girls are more exposed to gender-based violence, including sexual violence, during this period (24). A pandemic situation is something that affects overall functioning. Adolescents face worries that may overwhelm their emotional coping resources. Therefore, it is crucial to screen students with high levels of obsessive thoughts related to COVID-19 and give them immediate help.

### Current Study

Fear and obsession can increase the damage done by the disease itself. The emergence of COVID-19 (25) and its epidemic nature have exacerbated worldwide concerns that, in some cases, lead to stigma (26). A characteristic nature of infectious disease compared to other conditions is fear. Obsession and fear are directly related to the rate and environment of transmission (quickly and invisibly) and morbidity and mortality. With a high level of anxiety, individuals may not think clearly and rationally when reacting to COVID-19. Therefore, it is necessary to investigate the level of obsession with COVID-19 to provide better services for those involved. Adolescence, for which the consequences of a pandemic may have particularly negative emotional effects, deserve special attention. Therefore, we decided to test psychometric properties of the OCS (16) in Iranian adolescents.

Iran has been one of the first countries to report the outbreak of COVID-19 and has been since affected by the pandemic. Therefore, adolescents for a long time may feel tense and loneliness because they cannot spend time with friends as previously (5). Prolonged loneliness can lead to depression and anxiety disorders (13). The OCS (16) was the first measure developed to assess maladaptive coronavirus anxiety and obsession with the COVID-19. The OCS has been validated in the United States (16), Bangladesh (i.e., CAS) (27), Turkey (28), Pakistan (29), and Korea (30). It is unidimensional scale that consists of four items: “I had disturbing thoughts that I may have caught the coronavirus; I had disturbing thoughts that certain people I saw may have the coronavirus; I could not stop thinking about the coronavirus, and I dreamed about the coronavirus.” The research conducted so far indicated excellent psychometric properties of the OCS and international comparability of the results. As the psychological effects of the COVID-19 pandemic are observed worldwide, it is vital to expand the use of the scale in more countries. Since the pandemic affects people all over the world, it is possible to compare the feelings, beliefs and behaviors of people from different cultural contexts. Iran was one of the first countries to be affected by the virus and is still struggling with high number of mortality and social consequences, such as school closure. This study, which aimed to assess the psychometric properties of a scale measuring obsessive thoughts about a pandemic in Iranian adolescents, is an important contribution to the development of tools for individual diagnosis and comparative research.

Therefore, the study's main objective is to investigate the factor structure of the OCS in the Iranian sample. We tested the unidimensional factor model in accordance with previous findings. We were interested in testing the structure among the whole sample and checking whether there is the same structure regardless of the various group. Therefore, we tested measurement invariance across gender (girls, boys), inhabitancy (urban, rural), and infection experience (infected, non-infected). Finally, we calculated the reliability of the OCS in the whole sample and tested subgroups. We assumed that the OCS is unidimensional, the structure is comparable between various groups, and the scale has acceptable internal consistency. We also tested whether there are differences between various groups in the level of obsessive thought. We assumed that girls may have a higher score in the OCS than boys, that adolescents who were infected (or someone from their family members was infected) may report more obsessive thought than those from a non-infected group and there are no significant differences between adolescents from rural and urban areas.

## Methods

### Participants and Procedure

This study was part of a bigger project comparing Iran and Pakistan regarding COVID-19 stress and its effects on the adolescents population. For the purpose of this study, we used convenience cluster sampling. Students were selected from different parts of Markazi province, Iran, to include both rural and urban participants. Before the translation of the scale, author of the OCS was contacted by the first author to receive permission. After receiving the permission, the official steps of translating the scale were followed (explained in the measure section). Then, students were contacted through their school, and after receiving their parental consent, parents and adolescents were asked to complete an informed consent form. Then, students were able to complete the questionnaires using the link sent to their mobile phones. The questionnaires were designed using an Iranian online website called Porsline *(www.porsline.ir).* The link was sent to 1,000 students. After screening missing data, 709 students (girls *n* = 369, boys *n* = 339) were included in the study. We followed WHO definition of adolescence and include in this period students from 10 to 19 years old. Participants mostly aged 12–16 y.o., however, there were four students at age 11, 17, 18, and 19. All of them attended guidance school or high school. Recruitment of the participants was both from rural (*n* = 297) and urban (*n* = 412) Iran. To determine this, we asked the students if they were from the cities or the countryside. Among them, 131 people indicated that the student or one of the close family members had been infected COVID-19, 578 participants or their family members were not infected.

### Measures

#### Demographic Questionnaire

As the study was part of a larger project, participants were asked to complete a demographic questionnaire, which included the following questions: gender (52% girls, 48% boys), inhabitancy (42% rural, 58% urban), age, the device used for online classes (92% mobile phones, 8% tablet), internet type (100% mobile network), and whether COVID-19 has infected them or their family members (82% non-infected, 18% infected).

#### Obsession With COVID-19 Scale-Persian Version

The OCS is a recently developed, 4-point measure of persistent and disturbing thinking about COVID-19 that demonstrates solid reliability and validity using two U.S. samples (16). To translating the OCS, the scale was initially translated by two bilingual English translators. Both translators were experts in psychology (one M.A. and one Ph.D.). The agreement between the two translators was investigated to ensure inter-rater reliability. Inter-rater reliability is related to the stability of the translation reported by two or more translators from the same measurement (31). The original OCS scale was compared with the translated version, achieving acceptable inter-rater reliability among the two raters. Next, the final Persian translation was back-translated into English by bilingual Persian/English speaker and was approved by one of the authors, an expert in psychology and a fluent English speaker. Before main study, four students and one expert were asked to complete the questionnaire to see if the language was appropriative for their ages. Using a scale from 0 (not at all) to 4 (almost every day), respondents reported how often they thought persistent and disturbing about COVID-19 over the past 2 weeks. Higher scores indicate more obsessive thinking about COVID-19.

#### Methods of Data Analysis

To check whether the structure of the OCS was unidimensional as it was theoretically assumed, we used confirmatory factor analysis (CFA). The results were calculated in *lavaan* [R package (32)]. To evaluate the model-to-data fit, we applied common fit indices and evaluation criteria that indicate good model fit: χ^2^–non-significant, the root means the square error of approximation (RMSEA)—smaller than 0.08, the standardized root mean squared residual (SRMR)—smaller than 0.08, the comparative fit index (CFI)—above 0.95, and the Tucker-Lewis index (TLI)—above 0.95 (33, 34). Factor loadings (β) should be above the minimum recommended value > 0.40 (35).

Because the assumption of multivariate normality was violated, Mardia's test: skewness 2925.86, *p* < 0.001, kurtosis 66.10, *p* < 0.001, and variables were ordinal, we used the Diagonally Weighted Least Squares estimator [DWLS; (36)] to test structure of the OCS.

We applied the following criteria to evaluate measurement invariance models. We tested configural invariance in a model with no equality constraints imposed based on common model fit indices. We established metric invariance by fitting models where factor loadings on respective items were constrained to be equal across the groups. Finally, we examined scalar invariance based on constraint intercepts to be equal across the groups. To evaluate models, we used Chen's (37) recommendations: difference of fit indices between nested models in a large sample size (*N* > 300) should be smaller than 0.015 for RMSEA, 0.03 for SRMR, and 0.01 for CFI and TLI. We also applied the principle that the χ^2^/*df* ratio should be smaller than 3 to evaluate model as proper (38). To test internal consistency we assessed the Cronbach's alpha level with 95% *CI* and composite reliability [tested via online calculator (39)]. For all tests that we run, we adopted an alpha level of 0.05. The number of observations is sufficient to carry out the planned analyzes (40). Student's *t*-test and Mann-Whitney' *U* test were conducted to compare the OCS scores between groups (gender, inhabitancy, infection). Cohen's *d* was calculated to provide effect size for *t* and *U* test: 0.20 small effect, 0.50 medium effect, 0.80 large effect (41).

## Results

Descriptive statistics and the structure of the OCS were established. The unidimensional model that consists of four items obtained the required value of chi-square: $\chi_{\left(2\right)}^{2}=0.37,\;p=\;0.831$, *N* = 709, and the model-to-data fit indices were very satisfactory: RMSEA = 0 [0, 0.044, 90% *CI*], SRMR = 0.01, CFI = 1.00, and TLI = 1.00. All factor loadings were adequate because their values varied from 0.41 to 0.83. Item-scale Pearson's correlations varied from *r* = 0.49 to *r* = 0.85 (see Table 1) and indicate good properties of the scale.

**Table 1:** Descriptive statistics and CFA in the Iranian sample.

	**Item**	***M***	***SD***	**Range**	**Skew**	**Kurtosis**	**Item-scale *r***	**β CFA**
1	I had disturbing thoughts that I may have caught the coronavirus.	0.77	0.89	0–4	1.16	0.90	0.85	0.83
2	I had disturbing thoughts that certain people I saw may have the coronavirus.	0.91	0.97	0–4	1.07	0.70	0.84	0.77
3	I could not stop thinking about the coronavirus.	1.26	1.09	0–4	0.70	−0.28	0.83	0.69
4	I dreamed about the coronavirus.	0.10	0.39	0–3	4.62	24.64	0.49	0.41

*N = 709; all factor loadings and item-scale Pearson's r correlations are on the level p < 0.001*.

Then, we tested whether the OCS had the same measurement characteristics across all groups involved in the study (gender, inhabitancy, infection experience). Multivariate normality of the OCS in all groups was tested (see Table 2). The results indicated that the assumption was violated. Because the variables were ordinal, we applied the DWLS estimator in all tested models (36).

**Table 2:** Descriptive statistics and factor loadings in subgroups.

	**Girls**	**Boys**	**Urban**	**Rural**	**Non-infected**	**Infected**
**Descriptive statistics OCS**
*N*	369	339	297	412	578	131
*M*	0.82	0.70	0.78	0.74	0.75	0.81
*SD*	0.69	0.63	0.67	0.66	0.65	0.74
Range	0–3	0–3	0–3	0–3	0–3	0–3
Skewness	0.94	1.17	1.00	1.08	1.00	1.15
Kurtosis	0.29	1.26	0.50	0.86	0.60	0.73
**Multivariate normality (Mardia test)**
Skewness	1452.50^***^	1444.10^***^	1196.23^***^	1719.92^***^	527.94^***^	2441.20^***^
Kurtosis	43.14^***^	0.42^***^	41.76^***^	45.34^***^	22.91^***^	61.11^***^
***β***
OCS1	0.84	0.81	0.78	0.87	0.89	0.81
OCS2	0.78	0.75	0.79	0.76	0.75	0.77
OCS3	0.71	0.64	0.68	0.69	0.71	0.68
OCS4	0.44	0.43	0.35	0.45	0.61	0.35

*** *p < 0.001*.

First, measurement invariance across gender was established. The results indicated perfect model fit-to-the data for girls and boys. Moreover, in accordance with adopted criteria, equivalence on the configural, metric, and scalar level was fulfilled (see Table 3). All factor loadings were acceptable (>0.4, see Table 2). Then, we tested measurement invariance across inhabitancy. The results indicated an excellent model fit for adolescents from urban and rural areas. Additionally, the results confirmed multivariate invariance on the configural, metric, and scalar level (see Table 3). Although in urban children loading factor in OCS4 was slightly below the adopted criteria (β = 0.35), other loading factors were acceptable (>0.4, see Table 2). Finally, we tested measurement invariance across infection experience. The results indicated a very good model-fit-to-the-data for infected and non-infected children. It can be assumed that configural, metric, and scalar equivalence exists. Although we observed between configural and metric level ΔSRMR = 0.02, and according to assumptions, this change is slightly higher than criterion—should be <0.015—other criteria were fully met (see Table 3). In non-infected children we observed loading factor below 0.4 in OCS4 (β = 0.35), but others loading factors were acceptable (>0.4, see Table 2). Descriptive statistics for each group are presented in Table 2.

**Table 3:** Measurement invariance of the OCS across gender, inhabitancy, and infection groups.

	***N***	***df***	**χ^2^**	**RMSEA [90% *CI*]**	**SRMR**	**CFI**	**TLI**
**Gender**
Girls	369	2	0.08	0 [0–0]	0.007	1	1
Boys	339	2	0.65	0 [0–0.077]	0.023	1	1
Configural	–	4	0.72	0 [0–0.007]	0.013	1	1
Metric	–	7	1.47	0 [0–0]	0.017	1	1
Scalar	–	10	1.69	0 [0–0]	0.018	1	1
**Inhabitancy**
Urban	297	2	0.48	0 [0, 0.074]	0.019	1	1
Rural	412	2	0.09	0	0.008	1	1
Configural	–	4	0.57	0	0.01	1	1
Metric	–	7	1.09	0	0.013	1	1
Scalar	–	10	2.83	0	0.018	1	1
**Infection**
Infected	131	2	0.03	0	0.008	1	1
Non-infected	578	2	0.42	0 [0, 0.051]	0.012	1	1
Configural	–	4	0.45	0	0.01	1	1
Metric	–	7	4.89	0 [0, 0.052]	0.03	1	1
Scalar	–	10	5.11	0 [0, 0.028]	0.03	1	1

*In χ^2^ test all p > 0.05*.

Then, we compared groups. The results indicated that girls significantly exceed boys in the level of COVID-19 obsession thoughts: *t*_(706)_ = 2.35, *p* = 0.019 and difference is small (*d* = 0.18). There was no differences in the OCS score between adolescents from rural and urban areas: *t*_(707)_ = −0, 80, *p* = 0.422, *d* = 0.06 and groups of infected and non-infected: *U* = 36832.5, *p* = 0.628, *d* = 0.09. The results in all groups indicated right-skewed distribution, which means that most adolescents revealed a low level of obsession with COVID-19.

Summing up, results indicated that the OCS is a unidimensional scale that met the criteria for configural, metric, and full scalar invariance across gender (girls, boys), inhabitancy (urban, rural), and infection experience (infected, non-infected). Considering the full invariance of the OCS between groups, the descriptive statistics of the scale were calculated for the whole Iranian sample. The average level of OCS was weak (*M* = 0.76, *SD* = 0.66, *N* = 709, range 0–3), its distribution was right-skewed (skewness = 1.05, *W* = 0.90, *p* < 0.001) and close to mesokurtic (kurtosis = 0.69). The results in all groups indicated that most adolescents revealed a low level of obsession with COVID-19. Descriptive statistics for each group are presented in Table 2.

Finally, the reliability of the OCS was established for each group: Cronbach's α [95% *CI*] = 0.78 [0.74, 0.81] for girls, 0.75 [0.70, 0.79] for boys, 0.78 [0.74, 0.81] for rural children, 0.74 [0.69, 0.79] for urban children, 0.83 [0.81, 0.85] for infected with COVID-19, and 0.74 [0.66, 0.81] for those who were not infected. Composite reliability for the whole scale was 0.779.

## Discussion

The main objective of this study was to test the factor structure and reliability of the Obsession with COVID-19 Scale in an Iranian adolescents. The results confirmed unidimensionality of the OCS and its measurement invariance across gender, inhabitancy, and infections experience. Moreover, the Persian language version of OCS had a high internal consistency in entire sample and across various groups. The findings provided more evidence for a universality of the OCS structure presented by researchers from various countries (27–30). Thus, the Persian language version of the scale may contribute to further international research on the level of COVID-19 obsession.

Although the obsessive thoughts of COVID-19 disease is raised worldwide (42), the current study revealed relatively low level of obsessive thoughts about pandemic in Iranian adolescents. However, it doesn't mean that there are no adolescents who experience such obsessive thoughts. Students differ in the level of experienced COVID-19 thoughts, therefore OCS may be used as a screening tool that enables the observation of extremely high level of obsessive thoughts. There were no differences in the level of obsessive thought related to COVID-19 due inhabitancy and infection experience. It is likely that regardless of these factors, adolescents may count on appropriate help and access to medical care. However, girls reported more obsessive thoughts than boys. This result is consistent with previous findings that girls tend to report significantly higher depression and anxiety levels (43). Small differences between girls and boys may result from specific situation which is pandemic.

Our study has clear advantages and limitations. The OCS has only four items which facilitates its use in a short time. In the future, however, it can be consider modifying the scale and removing the last item. The fourth item had relatively lower psychometric properties compared to the other items, which is probably due to its separate content (related to dreams, not to daily thoughts). One of the limitations of our analysis is also that the infected/non-infected and rural/urban groups were unequal. Although measurement invariance in these groups is confirmed, it should be remembered that this group bias may influence the mean results of the tested population (especially the inhabitancy; infection is not something permanent).

Moreover, our sampling was not random and is limited to the group that we had access. We also did not controlled the language fluency and nationality of the participants. We tested adolescents who attended Iranian school and we assumed that all of them speak fluently in Persian language. This point is more than important because our study has all the disadvantages of self-report research (44). The results could be affected by temporary mood, level of mindfulness in reading items and instructions, the belief that participation in the study is important and makes sense, willingness to present themselves in a chosen way (45). The presented results should be treated as a starting point for further validation studies. The study was conducted only among adolescents which limits the possibility of generalizing the results to the whole Iranian population. Adults may experience more COVID-19 thoughts as they try to deal with own problems as well as resolve their children issues.

Moreover, this study was dedicated to establishing factor structure and reliability but convergent and divergent validity also should be tested. The future study should focus on relations between OCS to e.g. behaviors to avoid contamination or relations with obsessive-compulsive personality traits in adolescents and adults. Further studies should also check test-retest reliability to establish whether the responses are stable over time.

Diagnosis of pandemic related obsessive thoughts in adolescents is needed as the COVID-19 pandemic is still on going. As experts point out, it can be expected that the effects of the pandemic will be observed in the coming years (46). The period of adolescence is the time when young people establish relationships. During pandemic such developmental aim is difficult to meet. Pandemic as a global and extremely dynamic event has become a source of stress for billions of people around the world demanding unusual countermeasures (7). The lockdown led to the forced isolation of entire societies. A broadly defined lifestyle required change: the way we spend our free time, work and study (6). As this is the first time that modern generations have faced this kind of threat, it is important to test impact of pandemic on the young people mental health. As the pandemic is a global phenomenon, it is a special opportunity to check the impact of state policies on the mental health of citizens in different countries.

During crises, such as a global pandemic, seeking social support is often one of the most adaptable ways to deal with stress. Still, many government agencies have published guidelines on social distancing and hygiene. In other words, many of the recommendations to prevent the spread of COVID-19 include social distancing, which might prevent effective social support, which is required for well-being (47, 48). Many other strategies for dealing with stress, such as active coping with stress to alleviate problems, have also been reported to be effective during a crisis. Others, such as coping with substance use, have been found to be universally incompatible. Other strategies, such as distraction, might be context-dependent (49, 50). Prolonged restrictions of this type may have negative effects on mental health not only immediately but also in the long term. The use of a screening tool will help to prevent negative effects of pandemic on adolescents' future life.

## Conclusions

Summing up, the COVID-19 pandemic triggered physical, psychological, social, and economic impacts that have resulted in intense anxiety, depression, obsession, compulsion, etc. Adolescents are particularly vulnerable to emotional difficulties, therefore, it is crucial to support such group. To do so, it is essential to have reliable tools to diagnose the thoughts related to pandemic. It is possible thanks to the Persian version of the OCS which is a unidimensional and reliable scale. It can be dependably used for psychological research and individual diagnosis across various groups, including boys, girls, infected, non-infected, rural, and urban adolescents. As this study should be treated as a starting point for further studies, more data should be collected in the future to establish validity of the OCS in Iranian population.

## Data Availability Statement

The raw data supporting the conclusions of this article will be made available by the authors, without undue reservation.

## Ethics Statement

The studies involving human participants were reviewed and approved by Counseling Department Review Board in University of Isfahan, Iran. Written informed consent to participate in this study was provided by the participants' legal guardian/next of kin.

## Author Contributions

FA, MS, and AA contributed to the concept of the study. FA organized and conducted the research. MS performed the statistical analysis. All authors wrote the manuscript and accepted its final version.

## Funding

This work was supported by funds of the vice-rector of Pedagogical University of Krakow, granted to MS on the basis of the application number BN.302.257.2021.RN.

## Conflict of Interest

The authors declare that the research was conducted in the absence of any commercial or financial relationships that could be construed as a potential conflict of interest.

## Publisher's Note

All claims expressed in this article are solely those of the authors and do not necessarily represent those of their affiliated organizations, or those of the publisher, the editors and the reviewers. Any product that may be evaluated in this article, or claim that may be made by its manufacturer, is not guaranteed or endorsed by the publisher.
